# Sex- and cell-dependent contribution of peripheral high mobility group box 1 and TLR4 in arthritis-induced pain

**DOI:** 10.1097/j.pain.0000000000002034

**Published:** 2020-09-16

**Authors:** Resti Rudjito, Nilesh M. Agalave, Alex Bersellini Farinotti, Peter Lundbäck, Thomas A. Szabo-Pardi, Theodore J. Price, Helena Erlandsson Harris, Michael D. Burton, Camilla I. Svensson

**Affiliations:** a Department of Physiology and Pharmacology, Center for Molecular Medicine, Karolinska Institutet, Stockholm, Sweden; b Department of Neuroscience,Neuroimmunology and Behavior Group, School of Behavioral and Brain Sciences, University of Texas at Dallas, Richardson, TX, United States; c Department of Medicine, Center for Molecular Medicine, Karolinska Institutet, Stockholm, Sweden; d Department of Neuroscience, Pain Neurobiology Research Group, School of Behavioral and Brain Sciences, University of Texas at Dallas, Richardson, TX, United States

**Keywords:** HMGB1, Peripheral, Sex dimorphism, Hypersensitivity, Arthritis, TLR4

## Abstract

Spinal high mobility group box 1 protein (HMGB1) plays crucial roles in arthritis-induced pain; however, the involvement of peripheral HMGB1 has not been examined previously. In this study, we addressed the role of peripheral HMGB1 and explored if sex contributes differentially to nociception in arthritis. We found *Hmgb1* expression to be elevated in the ankle joints of male and female mice subjected to collagen antibody-induced arthritis. Blocking the action of peripheral HMGB1, however, only reversed collagen antibody-induced arthritis-mediated hypersensitivity in males. Intra-articular injection of the toll-like receptor (TLR)4-activating, partially reduced disulfide, but not the fully reduced all-thiol, HMGB1 evoked mechanical hypersensitivity in both sexes. A sex-dependent temporal profile in expression of inflammatory factors in the ankle joint was observed in response to intra-articular injection of disulfide HMGB1, with male mice showing a delayed, yet longer-lasting increase in mRNA levels for several of the investigated factors. Intra-articular HMGB1 did not induce cellular infiltration in the ankle joint suggesting its action on tissue resident cells. To further explore possible sex differences in cellular involvement, we used the macrophage inhibitor, minocycline, and mice with specific TLR4 depletion in myeloid cells or nociceptors. We found that inhibition of resident macrophages attenuated HMGB1-induced pain-like behavior only in male mice. Interestingly, although the contribution of TLR4 on myeloid cells to nociception was minimal in females compared to males, TLR4 on nociceptors are important for HMGB1-induced pain in both sexes. Collectively, our work highlights sex- and cellular location-dependent roles of HMGB1 and TLR4 in peripheral pain mechanisms.

## Abstract 2

Supplemental Digital Content is Available in the Text.

Peripheral high mobility group box 1 promotes joint pain through TLR4 activation in immune cells that is strongly evident in male but not female mice.

## 1. Introduction

High mobility group box 1 protein (HMGB1) is a nonhistone nuclear protein that can serve as an alarmin and plays key roles not only in inflammation but also in pain processing. Alarmins, including HMGB1, are evolutionary unrelated molecules that mediate divergent intracellular signaling, but once released due to cell death or inflammatory cues promote immune activation.^13,48^ The pronociceptive role of HMGB1 has been widely reported in the spinal cord, dorsal root ganglion (DRG), and local peripheral tissues in experimental pain models of rheumatoid arthritis (RA),^1^ neuropathy,^9,32,41^ osteosarcoma,^46^ interstitial cystitis,^23,27^ pancreatitis,^17,46^ and stroke.^12,30^ Interestingly, the actions of extracellular HMGB1 are dependent on its redox modifications. High mobility group box 1, released in the reduced all-thiol form, binds to receptor for advanced glycation end products (RAGE) and potentiates chemotaxis by forming a heterocomplex with CXCL12. During inflammation, all-thiol HMGB1 may be partly oxidized to the disulfide form, which acts on TLR4 and induces cytokine production.^13,48^ These interactions have been reported to occur in both male and female subjects.^1,56^

We have previously shown that spinal disulfide, but not all-thiol, HMGB1 induces pain-like behavior in male and female mice.^1^ Recent studies show that HMGB1 as a pain mediator is not only redox-dependent, but its actions may differ between sites. For example, intrathecal administration^1^ and bladder instillation^27^ of disulfide, but not all-thiol, HMGB1 causes pain-like behavior, whereas both isoforms cause excitation of DRG neurons^2^ and mechanical hypersensitivity after intraplantar injection.^53^ It is still not known, however, which redox form of peripheral HMGB1 is responsible for joint pain in RA. Joint pathologies in RA patients and animal models have been associated with elevated levels of HMGB1,^11,21,22,37,45^ whereas resident immune cells such as macrophages^14,45^ and neutrophils^1^ have been suggested as targets of HMGB1 in the joint. As joint compartments, except cartilage, are innervated by Aδ and C fibers^28^ and express TLR4^15^ and RAGE,^44^ a pressing question is through which mechanism HMGB1 released in the joint contributes to pain.

Increasing evidence indicates a sexually dimorphic role of TLR4 in pain. However, as the literature suggests, the contribution of TLR4 to nociception in rodents is rather complex. Despite both male and female mice expressing spinal TLR4, intrathecal injection of the TLR4 ligand, lipopolysaccharide (LPS) induces hypersensitivity only in male mice.^42^ By contrast, others have shown that LPS injection into the spinal cord as well as administration into the cerebral ventricle and hind paw produces allodynia in both sexes.^42,51^ The role of peripheral HMGB1 and TLR4 in pain has never been examined carefully in experimental arthritis with respect to sex-dependent mechanisms. Here, we used the collagen antibody-induced arthritis (CAIA) model to investigate if blockade of peripheral HMGB1 attenuates pain-like behavior in a sex-dependent fashion. Furthermore, we examined if intra-articular injection of HMGB1 induces mechanical hypersensitivity in both sexes, and through which receptors and cells HMGB1 exerts its actions.

## 2. Material and methods

### 2.1. Animals

All experiments were performed in accordance with protocols approved by the local ethical committees for animal experiments in Sweden (the Northern Stockholm Animal Ethical Committee) and the United States (the Institutional Animal Care and Use Committee of the University of Texas at Dallas), and were in accordance with the International Association for the Study of Pain guidelines. C57BL/6 and BALB/c male and female mice (10-12 weeks, 20-25 g) were purchased from Charles River (Freiberg, Germany) and Janvier (Le Genest-Saint-Isle, France). To generate cell-specific TLR4 depletion, TLR^fl/fl^ mice were used as previously described.^18^ TLR4^fl/fl^ mice were then crossed with mice expressing Cre under the control of the Na_v_1.8 or the lysozyme M (LysM) promoters to breed mice having TLR4 deletion in peripheral nociceptors or myeloid cells, respectively. The resulting LysM-TLR4^fl/fl^, Na_v_1.8-TLR4^fl/fl^, and TLR4^fl/fl^ (control mice) were backcrossed for 8 generations to a C57BL/6 background at the University of Texas at Dallas. Animals were housed in standard cages in animal facilities at Karolinska Institutet and the University of Texas at Dallas with 5 mice per cage, water and food ad libitum, pathogen-free environments, standard temperature, and a 12-hour light/dark cycle.

### 2.2. Collagen antibody-induced arthritis

The induction of CAIA in BALB/c mice and scoring of arthritis in the ankle joints were performed as previously described.^3^ In brief, mice were injected intravenously with an anti-collagen type II cocktail containing 5 monoclonal antibodies (Chondrex, Redmond, WA) on day 0 followed by intraperitoneal injection of 5 to 20 μg of LPS (Chondrex) on day 5. For scoring of arthritis, 1 point was given to every inflamed knuckle or toe, and a score of either 2.5 or 5 points was given to each paw, ankle, and wrist if moderately or severely inflamed, respectively. Each mouse had a maximum arthritis score of 15 points per paw and 60 points per animal. Mice that developed an arthritis score below 12 at the peak of inflammation were excluded from the study.

### 2.3. Drugs and drug delivery

Full-length endotoxin-free disulfide HMGB1 (HMGB1^C23-C45C106h^, disulfide HMGB1) was kindly provided by Dr. H. Yang (Feinstein Institute for Medical Research, Manhasset, NY) or purchased from HMGBiotech (Milan, Italy). In some experiments, the disulfide bridge between C23 and C45 was readily reduced by exposure to dithiothreitol (DTT, 5 mM) for 1 hour to generate fully reduced HMGB1 (HMGB1^C23hC45hC106h^, all-thiol HMGB1). The excess amount of DTT was removed by dialysis for 2 hours. High mobility group box 1 neutralizing antibody (2G7) was produced in house. Intra-articular injections were performed as described previously.^54^ Briefly, the mice were deeply anesthetized with 4% isoflurane in an induction chamber and maintained on 2.5% isoflurane through a nasal application. Injections of 1 μg disulfide or all-thiol HMGB1 (in 2.5 μL phosphate buffered saline [PBS]) were performed into the left intra-articular space in the ankle joint, whereas control groups were injected with 2.5 μL PBS. 2G7 was administered subcutaneously (100 μg/mouse, daily), whereas minocycline (30-100 μg/mouse; Sigma-Aldrich, St. Louis, MO) was coinjected with disulfide HMGB1 (1 μg/mouse) into the left ankle joint at a volume of 2.5 μL.

### 2.4. Behavioral tests

For measurement of mechanical hypersensitivity, mice were acclimatized and habituated to the test environment before baseline measurements. Three baselines were measured on 3 different days followed by randomization of the animals into different groups. Mechanical hypersensitivity was assessed by applying calibrated von Frey filaments (Marstock, Germany; and Stoelting Company, Wood Dale, IL) of incremental force to the plantar surface of the hind paw using the up-down method.^5^ Paw withdrawal thresholds were calculated as percentage change to the mean baseline values within each treatment groups. However, when there was a great variation of baselines between groups, withdrawal thresholds are presented as percentage change to individual baseline values. For intra-articular HMGB1-induced sensitization, mechanical hypersensitivity is presented as a hyperalgesic index (HI index), which is calculated from the area between the extrapolated baseline and the time–response curve after HMGB1 injection, and increasing values indicate increasing hypersensitivity. All behavioral experiments were performed during the day cycle by an experimenter who was blinded to the experimental conditions, such as drug treatments and genetically modified mice, throughout the experiment and data analysis to avoid bias.

### 2.5. Histology

Hind ankle joints were harvested from euthanized mice and postfixed in 4% paraformaldehyde solution (PFA, Histolab, Askim, Sweden) for 48 hours. Next, tissues were decalcified in 10% EDTA solution (Sigma-Aldrich) for 4 to 5 weeks, dehydrated in ethanol, and embedded in paraffin. Sagittal sections (5 μm) were stained with hematoxylin and eosin (Histolab) and examined using brightfield microscope (Nikon Eclipse TE-200, Tokyo, Japan).

### 2.6. Primary macrophage cell culture

Bone marrow cells were isolated from the tibia and femurs of BALB/c male or female mice, and cultured in low adherence T75 flasks (Sarstedt, Hildesheim, Germany) in macrophage medium. This medium contains Dulbecco's modified Eagle's medium (DMEM, Gibco, Carlsbad, CA) supplemented with 10% fetal bovine serum (Sigma-Aldrich, St. Louis, MO), 2 mM GlutaMAX, 50 U/mL penicillin, 50 μg/mL streptomycin (Gibco), and 10 ng/mL macrophage colony-stimulating factor (M-CSF, R&D System, Minneapolis, MN). Cells were cultured in a humidified incubator with 5% CO_2_ and upon reaching 75% to 80% confluency, cells were dissociated with 0.25% trypsin-EDTA (Sigma-Aldrich) and seeded at 1 x10^5^ cells/well in 96-well plate in serum-starved macrophage medium (0.5% fetal bovine serum) containing 1 ng/mL M-CSF. Cultured macrophages were stimulated in triplicates for each mouse with 1 μg/mL disulfide HMGB1 for 24 hours. The culture supernatants were then harvested for measurements of inflammatory factors.

### 2.7. Quantitative real-time polymerase chain reaction

Ankle joints (6 mm from each side of the joint capsules) of the hind legs and L3 to L5 DRG were dissected from euthanized mice and flash frozen. For ankle joints, frozen tissues were first crushed with a BioPulveriser (BioSpec, Bartlesville, OK) followed by bead homogenization using TissueLyser II (Qiagen, Hilden, Germany) in TRIzol reagent (Invitrogen, Carlsbad, CA). For DRG samples, frozen tissues were directly bead homogenized (Qiagen) in TRIzol reagent (Invitrogen). mRNA was extracted according to the manufacturer's protocol followed by cDNA synthesis using MultiScribe Reverse Transcriptase (Invitrogen). Quantitative real-time polymerase chain reaction was performed using StepOne Real-Time PCR Systems (Applied Biosystems, Foster City, CA) using hydrolysis probes to measure the relative mRNA levels. Predeveloped specific primer/probe sets for *Tnf* (Mm00443258_m1), *Ilb1* (Mm00434228_m1), *Il6* (Mm00446190_m1), *Ccl2* (Mm00441242_m1), *Cxcl1* (Mm04207460_m1), *Cxcl2* (Mm00436450_m1)*, Cox2 (Mm00478374_m1), Ngf (Mm00443039_m1), Cd34 (Mm00519283_m1), Mpo (Mm01298424_m1), Tlr2 (Mm00442346_m1), Rage (Mm01134790_g1), Cd11b (Mm00434455_m1), Hmgb1 (Mm00849805_gH), Tlr4* (Mm00445273), *Rplp2 (Mm00782638_s1)*, and *Hprt1* (Mm01545399_m1) were used for mRNA analyses (all from Applied Biosystems). Threshold cycle values for each sample were used to calculate the number of cell equivalents in the test samples using the standard curve method.^4^ The data were normalized to either *Rplp2* or *Hprt1* mRNA levels as housekeeping genes and expressed as relative expression or percentage change to the control groups.^20^ The effect of intra-articular injection was assessed in the injected joints collected 2, 4, and 6 hours postinjection, whereas basal mRNA expression was analyzed from noninjected ankle joints.

### 2.8. Electrochemiluminescence immunoassay

Levels of TNF, IL-6, CCL2, and CXCL1 were measured in macrophage culture supernatants using electrochemiluminescence immunoassay mouse kits (Meso Scale Discovery, Rockville, MD) following the manufacturer's instructions. Culture supernatants were diluted 1:2 in assay diluents. All electrochemiluminescence reactions were detected by MESO Quickplex SQ 120. The lower limit for quantification is 0.98 pg/mL for TNF, 7.61 pg/mL for IL-6, 4.42 pg/mL for CCL2, and 23.29 pg/mL for CXCL1.

### 2.9. Statistical analyses

Statistical tests were performed using GraphPad Prism 7 (San Diego, CA). To compare differences between groups split into 2 independent variables, two-way analysis of variance^49^ was used followed by Sidak or Tukey post hoc tests. To analyze differences between 2 groups, unpaired two-tailed Student *t* test was used, whereas comparisons of 3 groups or more was assessed using one-way analysis of variance followed by the Bonferroni post hoc test. All data are presented as mean ± SEM, and *P*-values less than 0.05 were considered statistically significant.

## 3. Results

### 3.1. Collagen antibody-induced arthritis increases gene expression of high mobility group box 1 in ankle joints of both male and female mice

**Figure 1.**
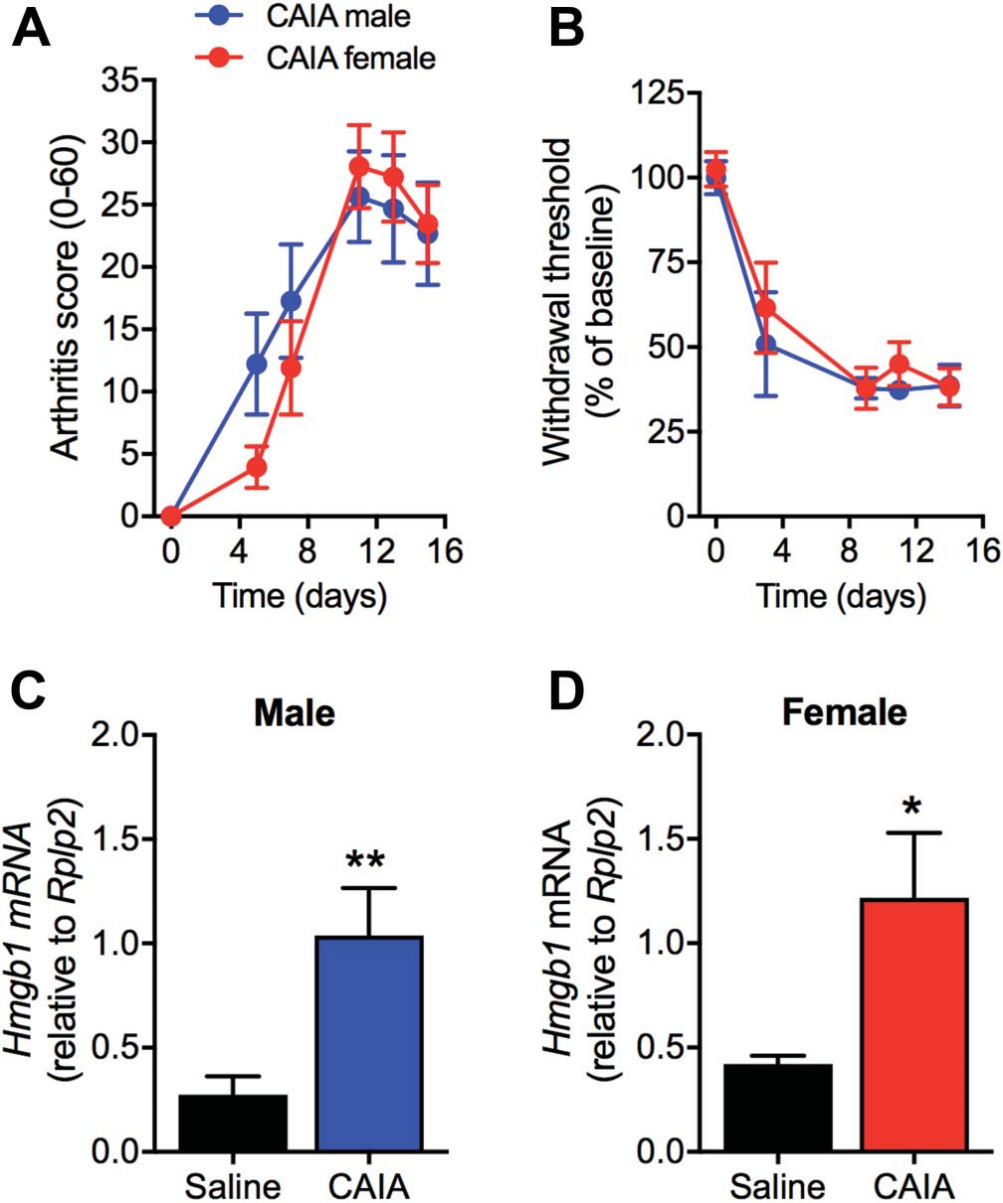
Collagen antibody-induced arthritis (CAIA) induces joint inflammation and mechanical hypersensitivity in both male and female mice and increases expression of *Hmgb1* mRNA in the ankle joints of the hind legs. (A) Scoring of arthritis assessed by visual inspection of the hind and front paws (score 0-60) and (B) mechanical hypersensitivity assessed by von Frey filaments for 16 days after CAIA induction. (C and D) *Hmgb1* mRNA levels in male and female ankle joints collected on day 16. Data are presented as mean ± SEM, n = 11 mice per group for arthritis scores and behavioral data, and n = 10 to 14 for mRNA data, *P < 0.05, **P < 0.01 vs saline group. HMGB1, high mobility group box 1 protein.

The CAIA model is associated with the development of joint inflammation and mechanical hypersensitivity in both male and female mice.^1,3^ In accordance with our previous reports, we did not observe any differences in disease severity or mechanical hypersensitivity between the sexes in mice that developed joint inflammation in this study (Figs. 1A and B). However, this notion is based on mice that developed arthritis. Mice that had arthritis scores less than 12 on day 12 were excluded from the study and, given these criteria, 35% males and 6% females were excluded. Thus, males showed a lower incidence of CAIA, as previously reported.^10^ *Hmgb1* mRNA levels were assessed in ankle joints of male and female mice 16 days after collagen type II antibodies injection. We found that *Hmgb1* mRNA levels were elevated in ankle joints of male (0.27 ± 0.09 vs 1.03 ± 0.22, n = 10-12, *P* = 0.003) and female (0.42 ± 0.04 vs 1.22 ± 0.31, n = 12-14, *P* = 0.027) CAIA mice compared to their respective control groups (Figs. 1C and D).

### 3.2. Blockade of peripheral high mobility group box 1 reverses collagen antibody-induced arthritis-mediated mechanical hypersensitivity in male but not female mice

**Figure 2.**
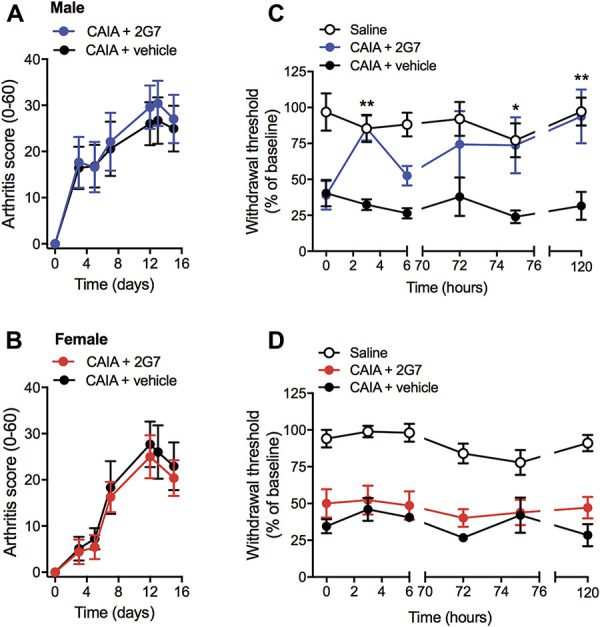
HMGB1 neutralizing antibody (2G7) reverses CAIA-induced mechanical hypersensitivity in male but not in female mice without affecting arthritis scores. (A and C) Arthritis scores and (B and D) mechanical hypersensitivity assessed in the CAIA model after subcutaneous injection of 2G7 (100 μg/mouse) or vehicle once a day from day 12 to day 16 in (A and B) male and (C and D) female mice. Data are presented as mean ± SEM, n = 6 mice/group, *P < 0.05, **P < 0.01 vs CAIA vehicle group. CAIA, collagen antibody-induced arthritis; HMGB1, high mobility group box 1 protein.

To examine if blocking the action of peripheral HMGB1 attenuated CAIA-induced joint inflammation and mechanical hypersensitivity, female and male mice were injected with 2G7, an HMGB1 neutralizing antibody^26^ (100 μg/mouse) once a day for 6 days starting from day 12. Although subcutaneous injection of 2G7 had no effect on the arthritis score of male or female mice (Figs. 2A and B), it significantly reversed CAIA-induced mechanical hypersensitivity at 3, 75, and 120 hours postinjection in male but not in female mice (Figs. 2C and D). Injection of the vehicle did not produce any effects at any timepoints.

### 3.3. Intra-articular injection of disulfide high mobility group box 1, but not all-thiol high mobility group box 1, induces pain-like behavior in male and female mice

**Figure 3.**
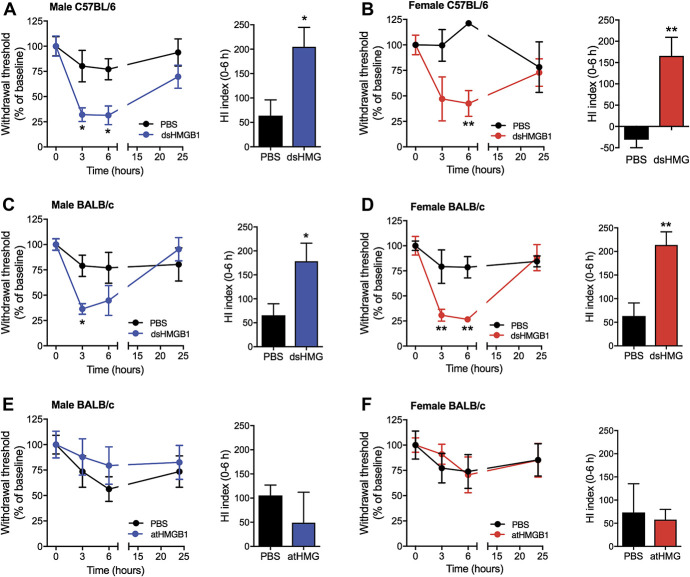
Intra-articular injection of disulfide HMGB1 (dsHMG), but not all-thiol HMGB1 (atHMG), into ankle joint induces mechanical hypersensitivity in male and female mice. Withdrawal threshold responses, assessed by von Frey filaments subsequent to intra-articular injection of dsHMG (1 μg/mouse), and HI indexes calculated for 0 to 6 h for C57BL/6 (A) male and (B) female and BALB/c (C) male and (D) female mice. Mechanical hypersensitivity and HI index (0-6 hours) induced by an intra-articular injection of all-thiol HMGB1 (1 μg) to BALB/c (E) male and (F) female mice. Data are presented as mean ± SEM, n = 4 to 8 mice/group, *P < 0.05, **P < 0.01 vs vehicle control group. HI, hyperalgesic index; HMGB1, high mobility group box 1 protein.

To investigate if the redox state of HMGB1 is critical for peripheral nociceptive signaling, and if there is an associated sex-dependence, disulfide or all-thiol HMGB1 (1 μg/mouse) was injected into the ankle joint of naive male and female mice and withdrawal thresholds were assessed over 24 hours. Intra-articular disulfide HMGB1 evoked mechanical hypersensitivity in male and female C57BL/6 (Figs. 3A and B) and BALB/c (Figs. 3C and D) mice, which lasted for at least 6 hours. Although some mice injected with PBS showed mild hypersensitivity in the ipsilateral paw, the mechanical thresholds were still significantly lower in the disulfide HMGB1 male and female groups (Figs. 3A–D). Calculation of the hyperalgesic index over 0 to 6 hours showed an increase in pain-like behavior of similar magnitude in male and female C57BL/6 and BALB/c mice receiving disulfide HMGB1 (Figs. 3A–D). By contrast, all-thiol HMGB1 did not evoke mechanical hypersensitivity in either male or female mice after intra-articular injection and did not lead to notable changes in the hyperalgesic index in any groups (Figs. 3E and F).

### 3.4. Intra-articular injection of disulfide high mobility group box 1 increases mRNA levels of several inflammatory factors at different timepoints in male and female joints

**Figure 4.**
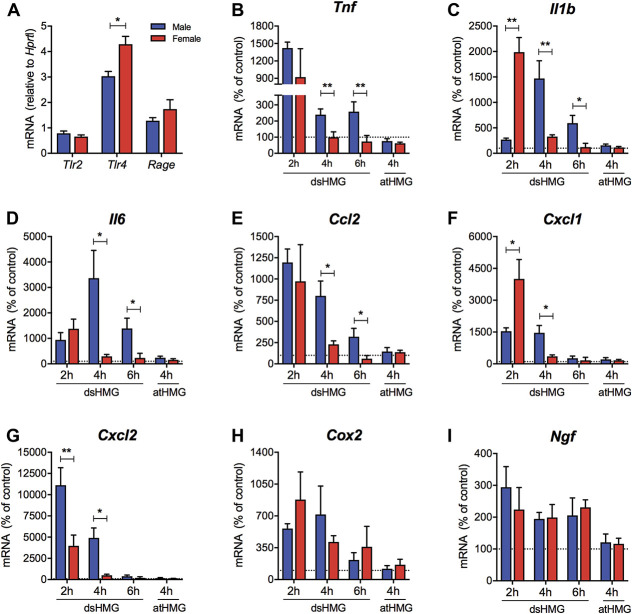
Intra-articular injection of disulfide HMGB1, but not all-thiol HMGB1, induces mRNA levels of inflammatory factors at different timepoints in ankle joints of male and female mice. (A) mRNA expression for HMGB1 receptors *Tlr2*, *Tlr4*, and *Rage* in noninjected ankle joints of males and female mice. mRNA expression for inflammatory factors (B) *Tnf*, (C), *Il1b,* (D) *Il6*, (E) *Ccl2*, (F) *Cxcl1*, (G) *Cxcl2*, (H) *Cox2*, and (I) *Ngf* in male and female mice injected with dsHMG or atHMG. Phosphate buffered saline-injected mice were used as vehicle control group and depicted as dashed lines in the graphs. Data are presented as mean ± SEM, n = 4 to 8 mice/group, *P < 0.05, **P < 0.01 male vs female. dsHMG: dsHMGB1, atHMG: atHMGB1. HMGB1, high mobility group box 1 protein.

We examined mRNA levels of *Tlr2*, *Tlr4*, and *Rage* (Fig. 4A) in noninjected ankle joints of male and female mice to indicate if the basal expression of HMGB1 receptors is sex-dependent. No differences in mRNA levels were detected for *Tlr2* and *Rage* between male and female mice. However, *Tlr4* expression was significantly higher in female compared to male mice (4.29 ± 0.31 vs 3.03 ± 0.16 REU, n = 5-6, *P* = 0.006). In addition, we measured basal expression of various inflammatory factors associated with HMGB1 action in noninjected ankle joints (Fig. S1A-F, available as supplemental digital content at http://links.lww.com/PAIN/B145). There were no differences in basal mRNA levels for these factors between male and female mice.

To examine if disulfide HMGB1 drives production of inflammatory factors in the joint, and if the degree of inflammatory response is similar in male and female mice, we extracted RNA from ankle joints 2, 4, and 6 hours after injection of disulfide or all-thiol HMGB1. Although injection of disulfide HMGB1 induced changes in gene expression, all-thiol HMGB1 did not elevate mRNA for any of the factors assessed in this study in either male or female ankle joints (Figs. 4B–I). After intra-articular disulfide HMGB1 injection, female mice showed robust mRNA induction of *Tnf*, *Il1b*, *Il6*, *Ccl2*, *Cxcl1*, and *Cxcl2* at 2-hour timepoint with *Il1b* and *Cxcl1* levels being higher compared to male mice at this timepoint (Figs. 4B–G). However, mRNA levels for most of these factors were not different compared to control mice by the 4-hour timepoint. By contrast, male mice showed more pronounced increase in mRNA levels of these factors starting at 4-hour timepoint, except for *Tnf* and *Cxcl2*, which had the highest levels at 2-hour timepoint (Figs. 4B–G). The levels of *Tnf, Il1b*, *Il6*, and *Ccl2* mRNA were significantly higher in males than females at 6-hour timepoint (Figs. 4B–E). Furthermore, the induction of *Cox2* and *Ngf* expression was similar in both male and female mice at all timepoints (Figs. 4H and I).

### 3.5. Intra-articular injection of disulfide high mobility group box 1 does not evoke cellular infiltration into the ankle joint

**Figure 5.**
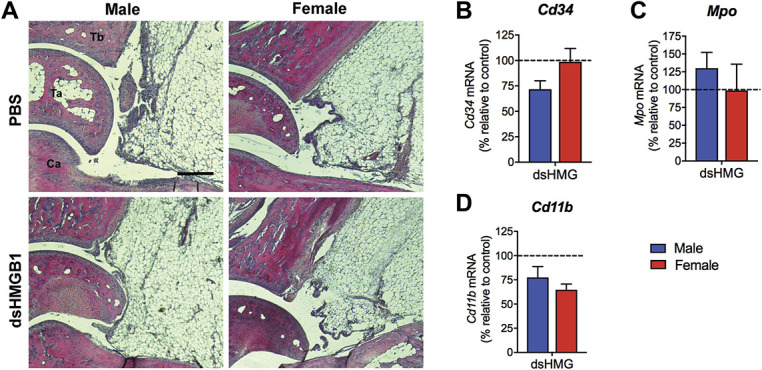
Injection of disulfide HMGB1 into the ankle joints does not induce local cellular infiltration. (A) Representative images of ankle joints stained by hematoxylin and eosin. Tb: tibia, Ta: talus, and Ca: calcaneus. Scale bar: 100 μm. mRNA expression for (B) *Cd34*, (C) *Mpo*, and (D) *Cd11b* in ankle joints of male and female mice injected with dsHMG. Phosphate buffered saline-injected mice were used as control group and depicted as dashed lines in the graphs. Data are presented as mean ± SEM, n = 5 to 6 mice/group. HMGB1, high mobility group box 1 protein.

The increase in cytokine and chemokine mRNA levels in the ankle joints could be due to infiltrating immune cells or activation of resident cells. Assessment of ankle joint sections stained with hematoxylin and eosin revealed no cellular infiltrate or structural lesions 4 hours after disulfide HMGB1 injection (Fig. 5A). To examine if there was a rapid increase in mRNA associated with inflammatory cells that may indicate infiltration or proliferation in the ankle joint, levels of *Cd34* (mast cells), *Mpo* (neutrophils), and *Cd11b* (macrophages) were assessed after disulfide HMGB1 injection. No significant increases in any of these factors were observed 4 hours after intra-articular injection (Figs. 5B–D).

### 3.6. Disulfide high mobility group box 1 stimulation induces higher levels of proinflammatory factors in macrophages derived from male compared to female mice

**Figure 6.**
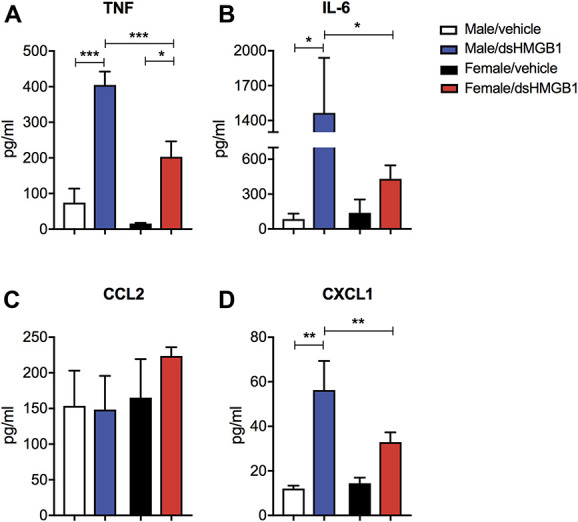
Addition of disulfide HMGB1 in culture induces higher levels of proinflammatory factors in male compared to female macrophages. Levels of (A) TNF, (B) IL-6, (C) CCL2, and (D) CXCL1 measured in culture supernatant after 24-hour stimulation of 1 μg/mL disulfide HMGB1 or phosphate buffered saline (vehicle control) of macrophages generated from male and female mice. Data are presented as mean ± SEM, n = 4 mice/group, *P < 0.05, **P < 0.01, ***P < 0.001. HMGB1, high mobility group box 1 protein.

We did not detect infiltration of immune cells into the ankle joint after disulfide HMGB1 injection, which may indicate that HMGB1 exerts its action on tissue resident cells, such as resident macrophages. Thus, we examined if disulfide HMGB1 induces the production of proinflammatory factors in primary macrophage culture and if the levels of these factors differ in cultures prepared from both male and female mice. We found a significant increase of TNF, IL-6, and CXCL1 levels in culture supernatant of macrophages derived from male mice after stimulation with disulfide HMGB1 (Figs. 6A–D). Only TNF levels were significantly elevated in female macrophages in response to disulfide HMGB1 exposure, whereas for IL-6 and CXCL1, we noted a trend towards increased release, albeit not statistically significant (Figs. 6A–D). The release of TNF, IL-6, and CXCL1 was, however, significantly higher in macrophage cultures from male compared to female mice (Figs. 6A–D). There was no difference in levels of CCL2 in cell culture supernatants after disulfide HMGB1 stimulation compared to the vehicle in either male- or female-derived macrophages (Fig. 6C).

### 3.7. Blockade of resident macrophages by minocycline protects male but not female mice from developing disulfide high mobility group box 1-induced pain-like behavior

**Figure 7.**
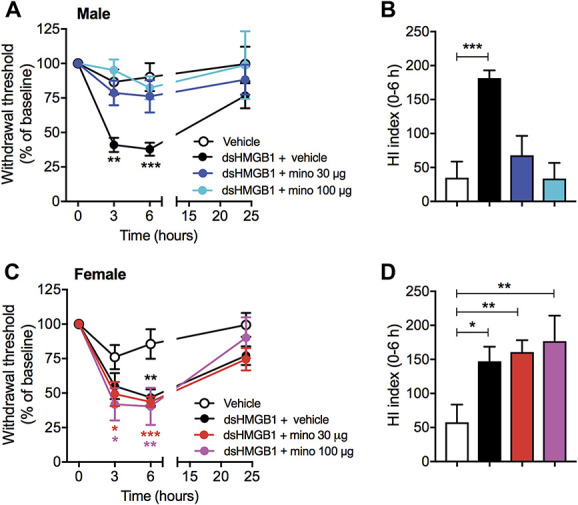
Inhibition of resident macrophages prevents male but not female mice from developing disulfide HMGB1-induced mechanical hypersensitivity. Withdrawal thresholds and HI indexes (0-6 hour) after intra-articular injection of disulfide HMGB1 (1 ug/mouse) in combination with either vehicle (phosphate buffered saline) or disulfide HMGB1 (1 ug/mouse) in combination with either vehicle or minocycline (30 ug and 100 ug/mouse) in (A and B) male and (C and D) female mice. Data are presented as mean ± SEM, n = 4 to 11 mice/group, *P < 0.05, **P < 0.01, ***P < 0.001 vs vehicle group. HI, hyperalgesic index; HMGB1, high mobility group box 1 protein.

Minocycline is a tetracycline antibiotic known to inhibit activation, proliferation, and cytokine-inducing activity of microglia and macrophages.^34^ Therefore, we examined if minocycline prevents disulfide HMGB1-induced pain-like behavior. Male and female mice were subjected to intra-articular injection to the ankle joint of either vehicle (PBS) or disulfide HMGB1 mixed with either vehicle or minocycline. Coadministration of disulfide HMGB1 and vehicle into the ankle joint induced mechanical hypersensitivity compared to the vehicle group, which was prevented when minocycline at both 30 and 100 μg was coadministered with HMGB1 in male mice (Figs. 7A and B). By contrast, none of the doses of minocycline produced antinociceptive effects in females. Female mice injected with minocycline together with HMGB1 exhibited reduction in paw withdrawal thresholds of similar magnitude as the disulfide HMGB1-vehicle group (Figs. 7C and D).

### 3.8. Disulfide high mobility group box 1-induced pain-like behavior is mediated by TLR4 on nociceptors for both sexes, but to a less extent on myeloid cells for females compared to males

**Figure 8.**
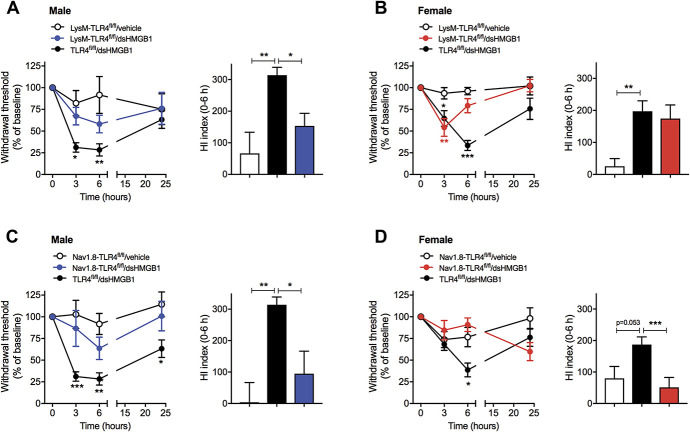
Depletion of TLR4 in nociceptors prevents HMGB1-induced hypersensitivity in male and female mice, whereas TLR4 depletion in myeloid cells protects male but not female mice from HMGB1-induced pain-like behavior. Withdrawal thresholds and HI indexes (0-6 hour) after intra-articular injection of disulfide HMGB1 (1 μg/mouse) or phosphate buffered saline (vehicle) in mice lacking TLR4 in (A and B) Na_v_1.8-expressing neurons (Nav 1.8-TLR4^fl/fl^) or (C and D) Lys-M-expressing myeloid cells (LysM-TLR4^fl/fl^) in (A and C) male and (B and D) female mice. Data are presented as mean ± SEM, n = 5 to 8 mice/group, *P < 0.05, **P < 0.01, ***P < 0.001 vs vehicle group. HI, hyperalgesic index; HMGB1, high mobility group box 1 protein.

To gain a better understanding of the contribution of macrophages in sex-dependent pronociceptive property of HMGB1, we used the conditional knockout mice that lack TLR4 in myeloid-derived cells (LysM-TLR4^fl/fl^) including monocyte, macrophages, neutrophils, and mast cells.^18^ Of note, we observed the temporal profile and degree of mechanical hypersensitivity to be different between male and female floxed mice (Figs. 8A and B). As such, intra-articular injection of disulfide HMGB1 to control (TLR4^fl/fl^) male mice led to a more pronounced mechanical hypersensitivity at the 3-hour timepoint compared to female TLR4^fl/fl^ mice. However, at the 6-hour timepoint, similar drops in paw withdrawal thresholds were noted between male and female TLR4^fl/fl^ mice. Assessment of behavior at the 3- and 6-hour timepoints after intra-articular injection of disulfide HMGB1 shows that LysM-TLR4^fl/fl^ male mice developed significantly less mechanical hypersensitivity compared to HMGB1 injected TLR4^fl/fl^ control mice at both timepoints. Female LysM-TLR4^fl/fl^ mice developed less mechanical hypersensitivity at the 6-hour time point compared to HMGB1-injected TLR4^fl/fl^ female mice (Figs. 8A and B). Calculation of the hyperalgesic index for the 0 to 6 h period shows that the overall consequence of TLR4 ablation in myeloid cells yields a statistically significant reduction of HMGB1-induced mechanical hypersensitivity in male but not female mice (Figs. 8A and B).

To further examine if disulfide HMGB1-induced nociception depends on TLR4 expressed on nociceptors and if there is a differential contribution of TLR4 expressed on these cells to hypersensitivity between male and female mice, we used mice lacking TLR4 in Na_v_1.8-expressing cells. For this, TLR4^fl/fl^ mice^18^ were crossed with mice expressing Cre recombinase under the Na_v_1.8 promoter. The resulting cross (Na_v_1.8-TLR4^fl/fll^) generates a 44% decrease in *Tlr4* mRNA expression in lumbar DRG (Fig. S2, available as supplemental digital content at http://links.lww.com/PAIN/B145). As previously described, injection of disulfide HMGB1 into female TLR4^fl/fl^ mice did not induce robust mechanical hypersensitivity compared to male TLR4^fl/fl^ or wild-type C57BL/6 female mice. TLR4 depletion in sensory neurons protected both male and female mice from developing disulfide HMGB1-induced hypersensitivity (Figs. 8C and D).

## 4. Discussion

In this study, we found that peripheral HMGB1 contributes to arthritis-induced pain. Although joint pain may be induced by local HMGB1 in a redox-dependent fashion in both male and female mice, CAIA-induced hypersensitivity is reversed by blocking peripheral HMGB1 in male mice only. Of importance, our data point to a more prominent role of immune cells in HMGB1-induced pain processing in male compared to female mice. Preventing activation of myeloid cells using pharmacological tools and genetically modified mice provided greater protection from developing HMGB1-induced hypersensitivity in males compared to females. By contrast, depletion of neuronal TLR4 attenuated HMGB1-induced pain signaling in both sexes. Together, our study demonstrates the important aspects of sex and cellular location in the contribution of peripheral HMGB1 and TLR4 to arthritis-induced pain.

High mobility group box 1 levels are elevated in the spinal cord after CAIA induction,^1^ but the levels of HMGB1 in the joint have not been previously examined. We therefore assessed ankle joints of mice subjected to CAIA and found that *Hmgb1* mRNA levels were induced to a similar degree in both male and female mice. We did not examine if HMGB1 was released or which cells in the joint express the protein. Previous work has shown that cytoplasmic HMGB1 protein is detectable in synovial macrophages and dendritic cells in male rats subjected to collagen-induced arthritis.^36^ Translocation of HMGB1 from nucleus to cytoplasm is an indicator of release, thus macrophages and dendritic cells are potential sources of HMGB1 in conditions with joint inflammation.

To further investigate the importance of HMGB1 in joint pain, we used an anti-HMGB1 neutralizing antibody (2G7) to block HMGB1 signaling in the CAIA model. Interestingly, although no apparent sex difference in pain-like behavior was observed in the CAIA model, blocking the action of peripheral HMGB1 with 2G7 only reversed mechanical hypersensitivity in male mice. The absence of an effect of 2G7 in female mice is surprising, given that the use of a systemic TLR4 antagonist prevents pain-like behavior in both male and female mice subjected to intraplantar formalin.^51^ Although the analgesic actions of drugs have most frequently been examined in male rodents, there are now an increasing number of studies addressing sex-dependent effects. Some of these show antinociceptive properties of certain substances to be more effective in females,^7,43^ whereas others have found signs of male-specific effects.^16,43^ Similar to our finding of a sex-dependent effect of 2G7, the specific underlying mechanism contributing to this disparity is far from clear and warrants further investigation.

Our previous work revealed a strong relationship between HMGB1 redox state and its pronociceptive properties in the spinal cord, but no distinguished sex-dependence was observed.^1^ This is rather remarkable, given the reports of sex dimorphism in the role of spinal TLR4 in pain signal transmission^42^ and therefore we examined if the role of peripheral HMGB1 is redox- and sex-dependent. We found that injection of disulfide, but not all-thiol, HMGB1 into the ankle joint induces mechanical hypersensitivity in both sexes. This finding is in agreement with prior work showing that intraplantar LPS, also a TLR4 ligand, induces mechanical hypersensitivity in both sexes.^42^ By contrast, both disulfide and all-thiol HMGB1 have been reported to induce hypersensitivity in male mice after intraplantar injection. It should be noted, however, that a 10-fold higher dose of all-thiol compared to disulfide HMGB1 was used to demonstrate this effect^53^ and because we only compared injection of the same dose of the 2 HMGB1 isoforms, we do not know if a larger amount of all-thiol HMGB1 would induce mechanical hypersensitivity. Our study thus shows that the ability of HMGB1 to induce nociceptive behavior after injection to the joint is redox-dependent but not sex-dependent.

Besides being widely known to be expressed in immune cells such as macrophages, TLR4 is also most likely to be present in nociceptors.^47,55^ Depleting TLR4 in Na_v_1.8-expressing nociceptors led to less pronounced HMGB1-induced mechanical hypersensitivity in both male and female mice, indicating that TLR4 expressed on nociceptors is important for HMGB1-induced nociception in both sexes. Because TLR4^fl/fl^ control female mice did not develop robust hypersensitivity at the 3-hour timepoint as compared to wild-type mice, this conclusion is largely based on the accumulated response over time (HI index) and therefore has to be viewed with caution. Of note, recent work also reported the contribution of neuronal TLR4 in pain-related behavior after nerve injury in both male and female mice.^52^ Thus, although HMGB1 is pronociceptive in both sexes and neuronal TLR4 seems to contribute to this, there may still be mechanistic aspects associated with differential actions on inflammatory cells that differ between sexes, in particular because it has been postulated that in the spinal cord, different nonneuronal cells contribute to sensitization in male and female mice, with microglia dominating this process in males and T cells in females.^43^ Recently, we also reported that spinal glial inhibition reversed mechanical hypersensitivity in male, but not female, mice in the postinflammatory phase of the CAIA model.^10^

We found that injection of disulfide HMGB1 led to elevated mRNA levels of inflammatory factors in the joint at different timepoints in male and female mice. Although the initial induction was more pronounced in females, most of these factors showed persistent increase in males at later timepoints. It has been shown that female mice exhibit a more efficient acute inflammatory response and that this is due to higher TLR expression in resident immune cells.^39^ Because we found basal *Tlr4* mRNA levels to be higher in females than males, this may account for the more robust initial response in females. However, as protein synthesis takes times, protein levels are normally associated with transcript changes at later timepoints^25^ as observed in male mice. Although we did not measure changes in the inflammatory factors at protein levels in the joint, our findings on cultured macrophages reveal that disulfide HMGB1 evoked higher protein production of some of these factors in males compared to females, suggesting a more prominent effect of HMGB1 on immune cells in males. These findings are in agreement with other reports showing that TLR4 activation induces greater inflammatory responses in male compared to female macrophages.^19,29^

Macrophages have been previously implicated in HMGB1-induced nociception,^40^ but not in the context of sex dimorphism. Because we did not observe cellular infiltration into the joint or increases in mRNA for markers of mast cells, neutrophils, or macrophages in either sexes after disulfide HMGB1 injection, this may indicate that HMGB1 acts on tissue resident cells in the ankle joint. The lack of signs of cellular infiltrate is not surprising because disulfide HMGB1 is not considered to be a strong chemotactic agent^54^ and it has been reported that HMGB1 injection into the joint induces cellular infiltration 4 days postinjection,^37^ whereas we only examined up to 6 hours postinjection. Because it has been shown that endogenous danger molecules, including HMGB1, do not directly induce activation of tissue resident mast cells,^8^ our data suggest that resident macrophages are the primary nonneuronal cellular target of HMGB1 in the male joint, in particular because local administration of minocycline and depletion of TLR4 on myeloid cells both reduced HMGB1-induced hypersensitivity to a greater extent in male compared to female mice. However, we cannot rule out the involvement of other cells because minocycline, in addition to inhibiting macrophage activity, also has effects on T cells and neurons,^31^ and the LysM promoter used for TLR4 deletion, besides being expressed in macrophages, is present also in other cells of the myeloid lineage.^57^

Epidemiological and clinical findings clearly demonstrate sex differences in pain conditions, with women at higher risk of developing chronic pain. However, the literature is still quite sparse when it comes to studies of sex-associated differences and behavior in rodent models of inflammatory pain. In the context of arthritis-induced pain, only few studies have made direct comparisons between male and female mice.^1,10,33,38^ Our current and previous studies^1,10^ show that CAIA induced a similar degree of hypersensitivity in both males and females. In agreement, no differences in pain-like behavior between sexes were reported after intradermal injection of Complete Freund's adjuvant^6^ and K/B x N serum transfer-induced inflammation.^50^ However, pain models of carrageenan-induced inflammation and colitis show more robust pain-like behavior in either males^24^ or females,^35^ respectively, when compared side by side. Therefore, further investigations are warranted to advance our understanding of which components of the inflammatory processes that generate pain are subject to sex dimorphism.

In conclusion, our current work shows that disulfide HMGB1 can promote joint pain through a peripheral mechanism of action in both male and female mice, and highlights the importance of cellular location as we found notable sex differences when it comes to immune cell-mediated pain mechanisms. We also provide evidence that TLR4 activation in immune cells contributes more strongly to pain in male mice, and may indicate that targeting this mechanism will be more effective for pain treatment in males with arthritis.

## Conflict of interest statement

The authors have no conflicts of interest to declare.

## Appendix A. Supplemental digital content

Supplemental digital content associated with this article can be found online at http://links.lww.com/PAIN/B145.
